# Dataset on the content of vitamin D_3_ and 25-hydroxyvitamin D_3_ in 40 pork (Breitov breed) commodities

**DOI:** 10.1016/j.dib.2022.108153

**Published:** 2022-04-09

**Authors:** Ali J. Othman, Ludmila G. Eliseeva, Fatima M. Duksi, Polina G. Molodkina, Alyona D. Shalankina

**Affiliations:** a Department of Commodity Research and Commodity Expertise, Plekhanov Russian University of Economics, Moscow, Russia; b Department of Natural Regenerated Resources and Ecology, Faculty of Agriculture, University of Aleppo, Syria

**Keywords:** Vitamin D_3_, 25-hydroxyvitamin D_3_, 25OHD_3_, HPLC, Pork, Food analysis

## Abstract

This dataset demonstrates the content of vitamin D_3_ and 25-hydroxyvitamin D_3_ (25OHD_3_) in boiled, grilled, and raw pork for both lean and whole cuts including ribs, tenderloin, shoulder, neck, belly, and center chops. Total fat content in raw pork cuts was determined by the gravimetric method. Quantification analysis using reverse phase high performance liquid chromatography with UV-DAD connected. Purification of fatty acids from proteins was performed using a c18 cartridge, resolving vitamin D_3_ from its metabolites by subjecting purified samples to polar silica cartridge, purification from sugars by a series of two amino columns, and quantification of vitamin D_3_ and 25-hydroxyvitamin D_3_ by injecting purified extracts on C18 SPE column. Grilling samples was conducted in an electric combi oven.

## Specifications Table

**Table:** 

Subject	Food Science: Food Chemistry
Specific subject area	Food analysis, vitamin D_3_ and 25-hydroxyvitamin D_3_ content in pork
Type of data	Table
How the data were acquired	Nexera X3 UHPLC 3.0 (Shimadzu, Japan) Electric combi oven (FlexFusionTM Henny Penny, USA) SPE column, CHROMABOND C18 (45 µm, 3 mL/500 mg, CHROMABOND®, Norcross, USA) SPE column,Silica cartridge Supelcosil LC-Si 5 (μm, 15.0 cm × 4.6 mm, Supelco®, Bellefonte, USA) SPE column, amino column Supelcosil (5 μm, 15.0 cm × 4.6 mm, Supelco®, Bellefonte, USA) SPE column RP C18 (Synergy hydro-RP 4,6 × 250 mm, 4.0 μm, Phenomenex Incorporation, USA) SPD-M20A Photodiode Array Detector (Shimadzu, Japan) ChromQuest 4.2.34 software for data collection and quantification The detection at 265 nm Pyrogallol (American chemical society grade) Petroleum ether (American chemical society grade, boiling range 24–24 C) Diethyl ether (HPLC grade) Potassium Hydroxide (KOH, HPLC grade) n-hexane (HPLC grade) Methanol (CH3OH, HPLC grade) Ethanol (C2H5OH, HPLC grade) Ascorbic acid (C6H8O6, American chemical society grade) Dichloromethane (CH₂Cl₂, HPLC grade) 2-propanol (C3H8O, HPLC grade) Cholecalciferol (vitamin D3) standard (HPLC grade) Ergocalciferol (vitamin D2) standard (HPLC grade) 25 Hydroxyvitamin D3 standard (HPLC grade) Hydrochloric acid (HCl, 8 M) Deerma Meat Grinder DEM-JR01 (Xiaomi Mi, China)
Data format	Raw and Analyzed data
Description of data collection	Pork samples used in this study were obtained from a 6 months old male pig bel to (Breitov breed) and had a carcass weight of 67.8 kg. Common lean and whole cuts (ribs, tenderloin, shoulder, neck, belly, center chops) were boiled, grilled, or kept raw and separated into lean and whole cuts. Lean samples: separated fats are removed and intermuscular fats were kept; Whole samples: are pork cuts without removing fat. The data on the contents of 25OHD_3_ and vitamin D_3_ were obtained by UHPLC according to Bilodeau et al. [1,2] with a slight modification. Pork boneless lean and whole cuts were grilled inside an electric combi oven (FlexFusionTM Henny Penny, USA). The data on the total content of fat in lean and whole cuts by the gravimetric method through solvent extraction after hydrochloric acid hydrolysis [3].
Data source location	Russian economic University. G. V. Plekhanova, Stremyanny avenue, 36, Moscow, 115093, Russia Institution: Plekhanov Russian University of Economics Latitude and longitude for collected samples: 55.7273, 37.6254
Data accessibility	1. With the article: Raw data (Supplementary material) Mean and the Standard Error of Mean (SEM) of the experimental data are available in this article in Tables (1 and 2) 2. Data repository: Repository Name: figshare Data identification number: 10.4121/17269247 Direct URL to data: https://figshare.com/s/e0c4a108530e7be1fcad

## Value of the Data

• This data is useful for positioning the primary contents of both vitamin D_3_ and 25OHD_3_ in different pork cuts.
• This data compares the content of vitamin D_3_ and 25OHD_3_ in lean and whole pork cuts subjected to boiling, grilling, and raw samples.
• This dataset provides additional information about 40 different pork commodities that would help fill the knowledge gaps regarding vitamin D_3_ and 25OHD_3_ contents of foodstuffs.
• This dataset can help nutritionists and nutrition-related applications in advising daily meal plans.

## 1 Data Description

Data provided in Table 1 was generated from 40 different pork commodities regarding the contents of vitamin D_3_ and 25OHD_3_ in lean and whole pork cuts subjected to boiling and grilling along with raw samples. Common cuts chosen for the analysis are: ribs, tenderloin, center chops, shoulder (blade), neck, belly (side), lean, and extra-lean grounded pork samples. Vitamin D_3_ and 25OHD_3_ were determined by HPLC with an analytical procedure as follows: (1) direct saponification; (2) extraction of the unsaponified partitions; (3) purification of the unsaponified fractions by C18, silica, and amino solid-phase extraction columns (SPE); (4) Quantification was performed by injecting purified extracts on RP C18 SPE column.

**Table 1:** Vitamin D_3_ and 25-hydroxyvitamin D_3_(mg/100 g; mean* (range; SEM)) in raw, boiled, and grilled pork commodities separated into lean and whole cuts.

Pork sample			Vitamin D3 (mcg/100 g)	25-hydroxyvitamin D₃ (mcg/100 g)	N*
Lean ground pork		raw	0.322 (0.31–0.346; 0.012)	0.326 (0.292–0.352; 0.018)	3
Lean ground pork		boiled	0.225 (0.22–0.23; 0.003)	0.173 (0.158–0.182; 0.007)	3
Extra-lean ground pork		raw	0.24 (0.23–0.251; 0.007)	0.222 (0.212–0.228; 0.005)	3
Extra-lean ground pork		boiled	0.13 (0.114–0.15; 0.011)	0.103 (0.092–0.125; 0.011)	3
Center chops	Lean	raw	0.245 (0.24–0.25; 0.005)	0.232 (0.221–0.247; 0.008)	2
Center chops	Lean	boiled	0.21 (0.2–0.22; 0.010)	0.226 (0.211–0.242; 0.009)	2
Center chops	Lean	grilled	0.202 (0.19–0.21; 0.008)	0.073 (0.068–0.074; 0.003)	2
Center chops	Whole	raw	0.346 (0.33–0.36; 0.014)	0.367 (0.341–0.393; 0.015)	2
Center chops	Whole	boiled	0.299 (0.318–0.28; 0.019)	0.247 (0.26–0.248; 0.008)	2
Center chops	Whole	grilled	0.231 (0.23–0.23; 0.001)	0.244 (0.232–0.254; 0.006)	2
Tenderloin	Lean	raw	0.049 (0.047–0.051; 0.001)	0.073 (0.07–0.079; 0.003)	3
Tenderloin	Lean	boiled	0.045 (0.042–0.45; 0.003)	0.04 (0.34–0.045; 0.003)	3
Tenderloin	Lean	grilled	0.019 (0.015–0.021; 0.002)	0.02 (0.017–0.022; 0.001)	3
Tenderloin	Whole	raw	0.208 (0.192–0.238; 0.015)	0.241 (0.224–0.265; 0.012)	3
Tenderloin	Whole	boiled	0.173 (0.169–0.177; 0.002)	0.196 (0.172–0.235; 0.020)	3
Tenderloin	Whole	grilled	0.111 (0.101–0.124; 0.007)	0.075 (0.072–0.81; 0.030)	3
Ribs	Lean	raw	0.1296 (0.126–0.136 0.003)	0.143	3
Ribs	Lean	boiled	0.104 (0.076–0.156 0.026)	0.106 (0.102–0.111; 0.003)	3
Ribs	Lean	grilled	0.0783 (0.067–0.096 0.009)	0.075 (0.065–0.089; 0.007)	3
Ribs	Whole	raw	0.183 (0.166–0.215; 0.016)	0.264 (0.258–0.269; 0.003)	3
Ribs	Whole	boiled	0.113 (0.101–0.127; 0.008)	0.138 (0.13–0.151; 0.007)	3
Ribs	Whole	grilled	0.091 (0.065–0.115; 0.014)	0.232 (0.192–0.262; 0.021)	3
Shoulder (blade)	Lean	raw	0.207 (0.202–0.212; 0.005)	0.288 (0.235–0.344; 0.032)	2
Shoulder (blade)	Lean	boiled	0.148 (0.142–0.154; 0.006)	0.165 (0.151–0.166; 0.008)	2
Shoulder (blade)	Lean	grilled	0.124 (0.127–0.136; 0.005	0.113 (0.111–0.115; 0.001)	2
Shoulder (blade)	Whole	raw	0.307 (0.295–0.32; 0.013)	0.337 (0.33–0.338; 0.004)	2
Shoulder (blade)	Whole	boiled	0.218 (0.23–0.206; 0.012)	0.226 (0.22–0.237; 0.005)	2
Shoulder (blade)	Whole	grilled	0.197 (0.194–0.201; 0.004)	0.137 (0.134–0.141; 0.002)	2
Neck	Lean	raw	0.07 (0.062–0.078; 0.008)	0.15 (0.41–0.167; 0.009)	3
Neck	Lean	boiled	0.071 (0.065–0.078; 0.007)	0.077 (0.064–0.089; 0.007)	3
Neck	Lean	grilled	0.042 (0.04–0.045; 0.003)	0.107 (0.072–0.12; 0.018)	3
Neck	Whole	raw	0.175 (0.17–0.181; 0.005)	0.232 (0.224–0.248; 0.008)	3
Neck	Whole	boiled	0.161 (0.16–0.162; 0.001)	0.205 (0.183–0.24; 0.018)	3
Neck	Whole	grilled	0.122 (0.12–0.125; 0.003)	0.081 (0.072–0.086; 0.004)	3
Belly (side)	Lean	raw	0.072 (0.067–0.08; 0.004)	0.105 (0.095–0.121; 0.008)	3
Belly (side)	Lean	boiled	0.081 (0.077–0.08; 0.003)	0.081 (0.064–0.09; 0.009)	3
Belly (side)	Lean	grilled	0.067 (0.065–0.71; 0.002)	0.094 (0.087–0.099; 0.004)	3
Belly (side)	Whole	raw	0.347 (0.324–0.376; 0.015)	0.338 (0.334–0.345; 0.003)	3
Belly (side)	Whole	boiled	0.334 (0.332–0.339; 0.002)	0.275 (0.27–0.283; 0.004)	3
Belly (side)	Whole	grilled	0.175 (0.241–0.262; 0.076)	0.18 (0.176–0.187; 0.004)	3

N*: number of repetitions.

Data provided in Table 2 demonstrates the total fat content in lean and whole raw pork cuts samples.

**Table 2:** Total fat content in lean and whole raw pork samples (wt%; range; SEM).

Pork sample		Total fat content
Lean ground pork	Lean	11.20 (10.25–12.74; 0.724)
Extra-lean ground pork	Whole	4.71 (4.25–5.61; 0.30)
Center chops	Lean	2.84 (2.38–3.21; 0.188)
Center chops	Whole	13.45 (12.38–114.88; 0.512)
Tenderloin	Lean	3.60 (3.39–3.82; 0.091)
Tenderloin	Whole	6.01 (5.67–6.65; 0.225)
Ribs	Lean	13 (12.12–13.81; 0.355)
Ribs	Whole	20.64 (17.23–22.92; 1.317)
Shoulder (blade)	Lean	13.6 (13.27–14.48; 0.293)
Shoulder (blade)	Whole	20.05 (18.08–21.6; 0.823)
Neck	Lean	3.78 (3.22–4.79; 0.346)
Neck	Whole	8.23 (6.87–9.35; 0.519)
Belly (side)	Lean	23.51 (21.63–25.53; 0.866)
Belly (side)	Whole	30.27 (28.58–31.63; 0.632)

Number of repetitions (*N* = 4).

## 2 Experimental Design, Materials and Methods

Pork meat used in this study was obtained from a male pig (Breitov breed), brought from the licensed slaughterhouse (Ariant, Moscow, Russia) on the 8th of November 2021 in when it was around 6 months old and had a carcass weight of 67.8 kg.

Common cuts were chosen for the analysis including ribs, tenderloin, shoulder (blade), neck, belly (side), and center chops. The mixture from all the previously mentioned cuts was used for making lean and extra-lean grounded pork samples. Bones were removed from all cuts, then cuts were placed in plastic containers provided with absorbent tray pads at the bottom and then frozen at −18 °C for 24 h before analysis.

### 2.1 Grilling

Dissection and separating of lean meat from connective tissues and adhering fat parts took place exactly before grilling. Pork boneless cuts were separated from each other's on metal trays and placed in a preheated Electric combi oven (FlexFusion^TM^ Henny Penny, USA). Samples were steamed for 30 min at a temperature of 220 °C.

### 2.2 Boiling

Each mass was cut into approximately 100 g pork cuts. Samples were obtained either by cubing the meat by cutting muscle fibers horizontally or slicing them by vertically cutting fibers. Samples were placed in a pan and boiled for 90 min with 1 L tap water (pH=7.18). No additives were added.

### 2.3 Analysis of 25OHD_3_ and vitamin D_3_

#### 2.3.1 Samples preparation

Analysis was performed in a closed laboratory without windows provided with special covered lamps. Nitrogen (N_2_) gas was used to replace air before the saponification step.

Ergocalciferol (vitamin D_3_) 99% and 25-hydroxyvitamin D_3_ (25OHD_3_) 99% (Merck KGaA, Darmstadt, Germany) both were of HPLC grades and served as standards.

Measures such as pre-cooling the sample were taken into account to eliminate the effect of the temperature rise during this procedure. Samples prepared for the test were analyzed without delay and protected from light.

#### 2.3.2 Saponification

A sample size of 50 g for each body part was thoroughly ground in a suitable mill and well mixed. Approximately 50 mg of the homogenized sample was transferred into a 10 ml glass test tube equipped with a proper cap. 3 mL of 96% Ethanol and 0.15 mL 95% KOH, 100 mL 1% pyrogallol, 0.25 mg ascorbic acid, 0.1 mL of Cholecalciferol 99% and 0.1 mL of Ergocalciferol 99% as internal standards were added and shaked for 2 min. The tube was transferred into 80 °C water bath and kept for 40 min. The cap was sealed up and 1 mL of bi-distilled water and 3 mL of n-hexane were added and then gently shaked.

The saponified mixture was centrifuged at 8000 rpm for 5 min. The top layer was collected and the extraction was performed again for better results with 3 mL of n-hexane without water and then centrifuges.

#### 2.3.3 Purification

Samples were washed in the hexane phase with 2 mL of bi-distilled water followed by centrifugation. The aqueous phase was separated and discarded. And 1 ml of 99.9% 2-propanol was added to the washed hexane phase. Samples were purified with solid-phase extraction (SPE) columns. The procedure was performed under a flow of nitrogen gas.

Purification of fatty acids of the samples from proteins was performed using a C18 cartridge (45 µm, 3 mL/500 mg, CHROMABOND®, Norcross, USA). Samples were subjected to a polar silica cartridge Supelcosil LC-Si 5 μm, 15.0 cm × 4.6 mm (Supelco, Bellefonte, USA) for resolving vitamin D_3_ and its metabolites in relevance to their polarity. Extracts were purified from sugars by connecting 2 amino columns (Supelcosil, 5 μm, 15.0 cm × 4.6 mm, Supelco®, Bellefonte, USA) in series for better separation.

#### 2.3.4 Quantification of vitamin D_3_ and 25-hydroxyvitamin D_3_

Elution was performed as the following: 12 mL of 0.4% (Dichloromethane/2-propanol) solution were added to 40 μm from the extracts of the SPE columns. Samples were evaporated under a stream of nitrogen gas until dry. The mixture was reconstituted with 350 μm of 0.4% (Dichloromethane/Isopropyl Alcohol) solution. Exactly 100 μL was injected at a flow rate of 1.0 mL/min in the device. 0.5% of (Isopropyl Alcohol/n-hexane) served as mobile phase.

Quantification of vitamin D_3_ and 25OHD_3_ was performed by injecting extracts on SPE column RP C18 (Synergy hydro-RP 4.0 μm, 4,6 × 250 mm, Phenomenex Incorporation, USA). Vitamin D_3_ and 25OHD_3_ were detected on a compacted UV photo-diode array at 264 nm. The content of Vitamin D_3_ was achieved from the relation among peak areas of both vitamin D_3_ and vitamin D_2_. An external standard calibration curve was used to calculate the content of 25OHD_3_ in pork samples.

### 2.4 Determination of total fat

The determination of total fat in pork samples was conducted by the gravimetric method [3]. Four samples (100 g) of each raw cut with rather similar fat concentrations were prepared for the analysis by grinding them using a meat grinder (DEM-JR01, Xiaomi Mi, China) and then storing them in plastic packs at a temperature of −20 °C for 12 h.

Each sample was treated with 8 M HCL, gently mixed, and ethanol was added. The liberated fat was then extracted with a mixture of petroleum ether and diethyl ether solvents. The solvent was evaporated and the fat was weighed then the percentage of fat content was calculated.

## Ethical Statement

There is no funding for the present effort. There is no conflict of interest. The data is available in public domain.

## CRediT authorship contribution statement

**Ali J. Othman:** Visualization, Methodology, Software, Formal analysis, Writing – original draft. **Ludmila G. Eliseeva:** Visualization, Formal analysis. **Fatima M. Duksi:** Formal analysis, Resources, Writing – original draft. **Polina G. Molodkina:** Methodology, Software, Formal analysis. **Alyona D. Shalankina:** Methodology, Software, Formal analysis.

## Declaration of Competing Interest

The authors declare that they have no known competing financial interests or personal relationships which have, or could be perceived to have, influenced the work reported in this article.

## Data Availability

Dataset on the content of vitamin D3 and 25-hydroxyvitamin D3 in 40 pork (Breitov breed) commodities (Original data) (figshare).

## Appendix Supplementary materials

Appendix A. Supplementary data Image, application 1
